# β-Selection: Abundance of TCRβ^–^/γδ^–^ CD44^–^CD25^–^ (DN4) cells in the foetal thymus

**DOI:** 10.1002/eji.200636503

**Published:** 2007-02

**Authors:** Ariadne L Hager-Theodorides, Nicola J Rowbotham, Susan V Outram, Johannes T Dessens, Tessa Crompton

**Affiliations:** 1 Division of Cell and Molecular Biology, Faculty of Natural Sciences, Imperial College LondonLondon, UK; 2 Department of Infectious and Tropical Diseases, London School of Hygiene and Tropical MedicineLondon, UK

**Keywords:** β-Selection, Differentiation, DN3/4, Pre-TCR, Thymocyte

## Abstract

Expression of TCRβ and pre-TCR signalling are essential for differentiation of CD4^–^CD8^–^ double negative (DN) thymocytes to the CD4^+^CD8^+^ double-positive (DP) stage. Thymocyte development in adult Rag1, Rag2 or TCRβδ-deficient mice is arrested at the DN3 stage leading to the assumption that pre-TCR signalling and β-selection occur at, and are obligatory for, the transition from DN3 to DN4. We show that the majority of DN3 and DN4 cells that differentiate during early embryogenesis in wild-type mice do not express intracellular (ic) TCRβ/γδ. These foetal icTCRβ^−^/γδ^−^ DN4 cells were T lineage as determined by expression of Thy1 and icCD3 and TCRβ DJ rearrangement. In addition, in the foetal Rag1^–/–^ thymus, a normal percentage of DN4 cells were present. In wild-type mice after hydrocortisone-induced synchronisation of differentiation, the majority of DN4 cells that first emerged did not express icTCRβ/γδ, showing that adult thymocytes can also differentiate to the DN4 stage independently of pre-TCR signalling. Pre-TCR signalling induced expansion in the DN4 population, but lack of TCRβ/γδ expression did not immediately induce apoptosis. Our data demonstrate *in vivo* differentiation from DN3 to DN4 cell in the absence of TCRβ/γδ expression in the foetal thymus, and after hydrocortisone treatment of adult mice.

## Introduction

Signalling through the pre-T cell receptor (TCR) is essential for differentiation of CD4^–^CD8^–^ double-negative (DN) thymocytes to the CD4^+^CD8^+^ double-positive (DP) stage [1, 2]. The DN population can be sub-divided into four subsets, DN1–DN4, depending on the expression of the cell surface molecules CD44 and CD25. The earliest CD44^+^CD25^–^ (DN1) cells give rise to the CD44^+^CD25^+^ (DN2) population that progresses to the CD25^+^CD44^–^ (DN3) stage, and then to the CD44^–^CD25^–^ (DN4) population [3]. Expression of rearranged TCRβ and formation of the pre-TCR complex leads to a series of events collectively known as β-selection, resulting in allelic exclusion of the TCRβ locus, expansion and differentiation to the DP stage [4]. Several studies have indicated that thymocytes that successfully pass the β-selection checkpoint receive pre-TCR induced signals for proliferation [4–6] and survival [7, 8]. Recently, BCL2A1 has been identified as an anti-apoptotic agent that is up-regulated following pre-TCR signalling [9].

Rearrangement of the TCRβ locus and expression of a functional TCRβ chain are essential for differentiation, and the development of thymocytes in adult mice deficient in Rag1 [10], Rag2 [11] or TCRβδ [12] is arrested at the DN3 stage. The fact that T cell development in these mice is arrested at the DN3 stage has lead to the assumption that pre-TCR signalling and β-selection occur at the transition from DN3 to DN4 and are obligatory for differentiation beyond the DN3 stage of development. Contrary to this assumption, rare DN4 cells that do not express TCRβ or γδ have been detected in adult wild-type mice [7]. As cell death is increased in this TCRβ^–^/γδ^–^ DN4 population, it has been suggested that cells that fail β-selection die at the DN4 stage [7]. Here we show that in the wild-type foetal thymus differentiation from DN3 to DN4 frequently occurs independently of TCRβ/γδ expression (and hence normal pre-TCR signalling).

In addition, in the foetal Rag1^–/–^ thymus a normal percentage of DN4 cells were present, although no DP cells were observed. In the foetal thymus, T cell development occurs in a largely synchronized wave [13], allowing the study of thymocyte populations as they first emerge, and enabling the definition of requirements for the transition of cells from one population to the next. After synchronizing adult thymocyte development by hydrocortisone treatment, the majority of DN4 cells that emerge *in vivo* in the adult thymus do not express intracellular (ic) TCRβ/γδ.

## Results

### The majority of early foetal DN4 thymocytes do not express icTCRβ or γδ

**Figure 1:**
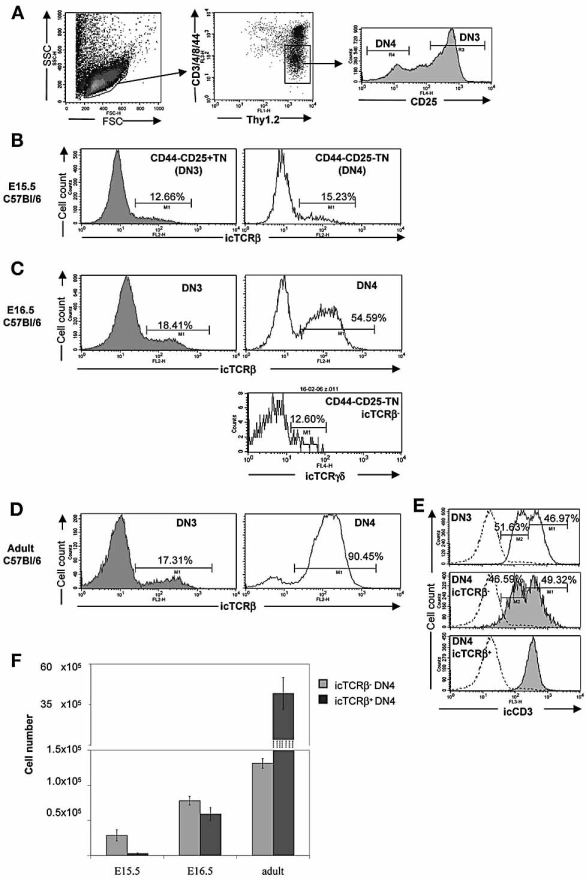
Analysis of DN3 and DN4 foetal and adult wild type thymocytes. (A) Gates for the identification of DN3 and DN4 subsets (shown for E16.5 thymus). Thymocytes in the live FSC/SSC gate (left) were determined as DN3 or DN4 when falling in the gate negative for anti-CD3/CD4/CD8/CD44 and positive for anti-Thy1.2 antibody staining (middle) and positive or negative for anti-CD25 staining (right), respectively. (B–D) Percentages of icTCRβ^+^ cells among CD44^–^CD25^+^ TN (DN3) and CD44^–^CD25^–^ TN (DN4) subsets are shown for E15.5 (B), E16.5 (C) and adult thymus (D). The percentage of icTCRγδ^+^ cells in the icTCRβ^–^ DN4 subset on E16.5 is also shown (C). (E) Expression of icCD3 in DN3, icTCRβ^–^ and icTCRβ^+^ DN4 thymocytes. Expression of icCD3 in DN3 cells is shown in the top, icTCRβ^–^ DN4 in the middle and icTCRβ^+^ DN4 cells in the lower panel. Percentages of high and low icCD3 expression are shown for the DN3 and icTCRβ^–^ DN4 subsets. The dotted line in all histograms represents the expression of icCD3 in B lymphocytes, similar in size to thymocytes and isolated from lymph nodes of adult wild-type mice, and is a negative control for the icCD3 staining. (F) Kinetics of icTCRβ^–^ and icTCRβ^+^ DN4 thymocyte number. The number of icTCRβ^–^ DN4 and icTCRβ^+^ DN4 thymocytes are shown for E15.5, E16.5 and adult thymi. Bars represent the mean of at least three thymi and error bars the standard error of the mean.

Analysis of TCRβ expression in foetal thymocytes revealed a large population of CD3^–^CD4^–^CD8^–^ triple negative DN4 cells (CD44^–^CD25^–^) that did not express detectable icTCRβ. We analysed the intracellular expression of TCRβ in DN3 and DN4 cells of embryonic day (E) 15.5, E16.5 and adult wild-type thymi. DN3 and DN4 subsets that fell within the live FSC/SSC gate were identified by positive staining for anti-Thy1.2, presence or absence, respectively, of cell surface expression of CD25 and the absence of CD44, CD3, CD4, CD8 expression (Fig. 1A). In a typical experiment, on E15.5, 12.66% of the DN3 cells and 15.23% of the DN4 cells expressed icTCRβ (Fig. 1B). The percentage of icTCRβ^+^ cells increased in both DN3 and DN4 subsets on E16.5 and in a typical experiment 18.41% of the DN3 and 54.59% of the DN4 cells expressed icTCRβ (Fig. 1C). On E16.5, approximately 12% of icTCRβ^–^ DN4 cells stained positively for icTCRγδ expression (Fig. 1C). Thus, 40% of DN4 cells on E16.5 did not express either icTCRβ or TCRγδ. As previously shown, in the adult thymus, 18.49% of DN3 and 90.45% of DN4 cells expressed icTCRβ (Fig. 1D).

The fact that the majority of the E15.5 foetal DN4 thymocytes were icTCRβ^–^ was surprising as, in contrast to the adult thymus [5, 7, 14, 15], in the foetal E15.5 thymus there was no enrichment for icTCRβ^+^ cells in the DN4 population compared to the DN3 subset (Fig. 1), suggesting that the foetal TCRβ^+^ DN3 cells did not preferentially differentiate to the DN4 stage compared to the foetal TCRβ^–^ DN3 cells. Analysis of icCD3 expression confirmed that the icTCRβ^–^ DN4 cells were T lineage cells. Both icTCRβ^–^ and icTCRβ^+^ DN4 cells expressed icCD3 and 54% of the icTCRβ^–^ and 90% of the icTCRβ^+^ DN4 cells expressed icCD3 at high level (Fig. 1E).

Given the reduction in the proportion of icTCRβ^–^ DN4 cells present in the thymus from E15.5 to E16.5 to adult, it seemed likely that icTCRβ^–^ DN4 cells are gradually removed from the thymus from E15.5 onwards. We therefore calculated the absolute numbers of icTCRβ^–^ and icTCRβ^+^ DN4 cells in the thymus on E15.5, E16.5 and in adults. We found that the number of icTCRβ^–^ DN4 thymocytes in fact increases over time but to a lesser extent than the number of icTCRβ^+^ DN4 cells, accounting for the reduction in overall percentage (Fig. 1F).

### DN4 cells develop in Rag1^–/–^ foetal thymus

**Figure 2:**
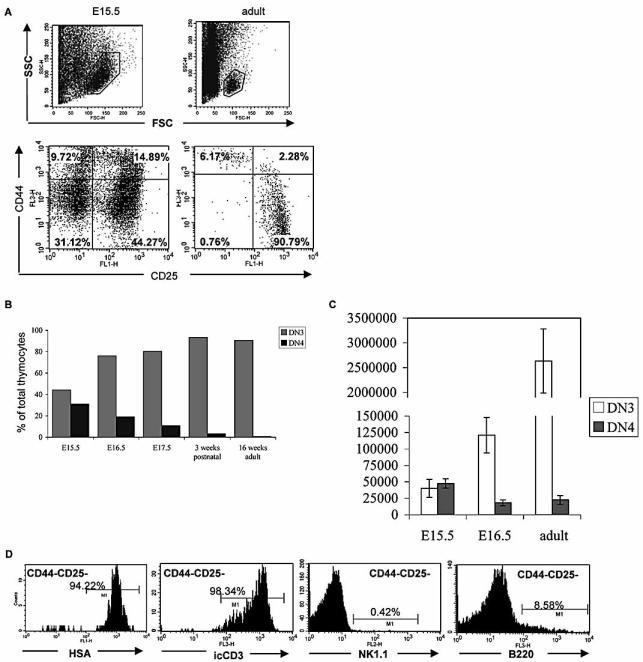
Analysis of Rag1^–/–^ thymocyte subsets. (A) CD44 and CD25 expression in E15.5 and adult Rag1^–/–^ thymocytes. Top panel are FSC/SSC dot plots profiles of E15.5 and adult thymocytes; live gates are shown. Profiles in the lower panel show cells that fell in the live gate and were negative for anti-CD3/CD4/CD8 antibody staining. CD44 against CD25 dot plots and percentages of cells in each quadrant are given for E15.5 (left) and adult (right). (B) Graph representing the percentages of thymocytes in the DN3 and DN4 subsets in E15.5, E16.5, E17.5, juvenile (3-week-old) and adult Rag1^–/–^ mice. (C) Absolute number of DN3 and DN4 cells in the E15.5, E16.5 and adult Rag1^–/–^ thymi. Bars represent the mean of at least three mice. Cells were gated positive for Thy1.2 staining, negative for CD4, CD8 and CD44 expression and positive or negative for CD25 surface expression, respectively. (D) Expression of lymphocyte markers in E15.5 DN4 Rag1^–/–^ thymocytes. Histograms show the expression of HSA, icCD3, NK1.1, B220 in CD44^–^CD25^–^ cells and the percentage of CD44^–^CD25^–^ cells positive for each marker are shown. Cells were gated negative for CD25, CD44, CD3, CD4 and CD8.

The presence of icTCRβ^–^ DN4 cells in the first wave of differentiation of wild-type foetal thymocytes led us to ask whether DN4 cells are present in Rag1^–/–^ foetal thymi. We analysed Rag1^–/–^ foetal thymi at E15.5, E16.5, E17.5, thymi at 3 weeks postnatally (juvenile) and adult thymi and observed a substantial DN4 population on all embryonic days tested (Fig. 2A, B), whereas the percentage of DN4 thymocytes was extremely small in the adult thymus of Rag1^–/–^ mice as previously reported [10]. In a typical experiment, 31.12% of the thymocytes were DN4 on E15.5 compared to only 0.76% of the adult thymocytes. The percentage of DN4 thymocytes gradually decreased over time until they were virtually undetectable in the adult thymus (Fig. 2B). Correspondingly, the percentage of DN3 cells increased substantially from E15.5 to E16.5 and thereafter the thymus consisted mainly of DN3 thymocytes (Fig. 2B). As expected, no TCRβ expression or DP thymocytes were observed at any stage of embryonic or postnatal development in Rag1^–/–^ mice (data not shown). Although the percentage of Rag1^–/–^ DN4 thymocytes gradually decreased over time after E15.5, the absolute number of DN4 cells did not change appreciably, whereas the number of DN3 cells increased (Fig. 2C).

The Rag1^–/–^ DN4 cells observed, as defined by the absence of CD44 and CD25 cell surface markers, were true T lineage DN4 cells as the majority stained positive for cell surface expression of HSA (94.22%) and expressed intracellular CD3 (Fig. 2D). In contrast, in a typical experiment only 0.42% stained positive with anti-NK1.1 and 8.58% with anti-B220 antibodies (Fig. 2D).

### Transcription of Rag1 and Rag2 is down-regulated in the foetal DN4 population

**Figure 3:**
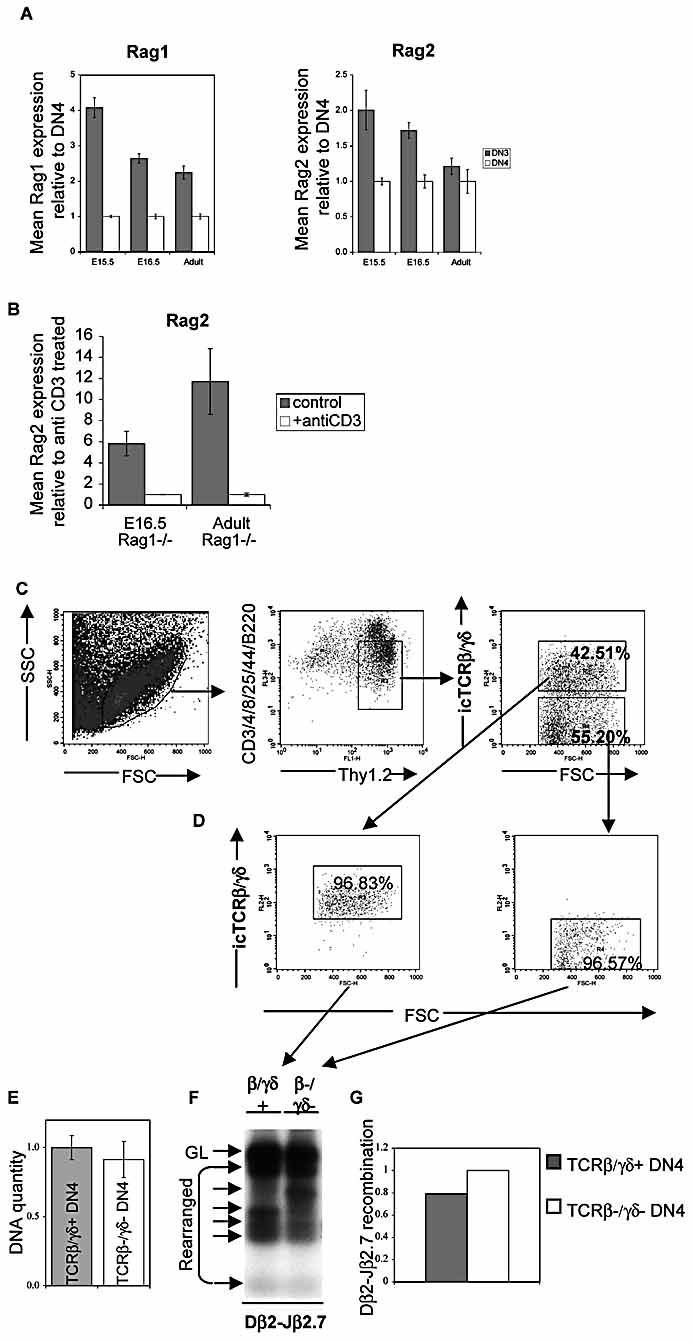
Transcription of Rag1 and Rag2 genes in foetal and adult DN3 and DN4 thymocyte subsets and TCRβ locus rearrangement. (A) Relative transcription of Rag1 (left graph) and Rag2 (right graph) in DN3 (full bars) and DN4 (open bars) sorted thymocytes from E15.5, E16.5 and adult wild-type thymi. DN3 and DN4 cells were sorted to be positive for Thy1.2 expression, negative for CD3, CD4, CD8, CD44 and B220 expression and positive or negative for CD25 expression, respectively. (B) Transcription of Rag2 in Rag1^–/–^ E16.5 and adult thymi, cultured for 24 h in the presence or absence of anti-CD3ε antibody is shown for control (full bars) and treated (open bars) cultures. (C–G) Dβ2-Jβ2.7 rearrangement of the TCRβ locus in TCRβ^–^/γδ^–^ and TCRβ/γδ^+^ DN4 wild-type E16.5 foetal thymocytes. TCRβ^–^/γδ^–^ and TCRβ/γδ^+^ DN4 foetal thymocytes falling in the FSC/SSC live gate (C, left) were sorted negative for CD3/4/8/25/44/B220 and positive for Thy1.2 (C, middle) and negative or positive for icTCRβ/γδ staining, respectively (C, right). Purity of the sorted populations was confirmed by the icTCRβ/γδ expression profiles shown (D). Histogram (E) shows the quantification of the TCRβ^–^/γδ^–^ and TCRβ/γδ^+^ DN4 thymocyte DNA preparations as assessed by real time PCR. Southern hybridisation was performed on PCR products amplified using specific Dβ2 and Jβ2.7 primers from equal amounts of DNA preparations from each thymocyte subset (F). The probe used for the southern hybridisation corresponded to the full-length, germline (GL) TCRβ locus. Quantification of the extent of Dβ2-Jβ2.7 rearrangement relative to the TCRβ^–^/γδ^–^ thymocytes, calculated for each cell type as the ratio of the intensity of the band corresponding to the GL sequence divided by the total intensity of the bands corresponding to the six rearranged TCRβ, is also shown (G).

It has been shown that transcription of Rag1 and to a lesser extent Rag2 is down-regulated in adult DN4 cells relative to DN3 cells [16]. To test whether down-regulation of the Rag genes in the DN4 relative to the DN3 population depended on the expression of TCRβ/γδ, we analysed the transcription of Rag1 and Rag2 by quantitative RT-PCR in DN3 and DN4 thymocytes sorted from E15.5, E16.5 and adult wild-type thymi. Rag1 transcripts were two to four times less abundant in DN4 thymocytes compared to DN3 cells (Fig. 3A, left panel). More than 80% of DN4 thymocytes on day E15.5 did not express TCRβ, suggesting that down-regulation of Rag1 in the DN4 subset on day E15.5 was independent of pre-TCR signalling. Rag2 transcription was also significantly decreased in foetal DN4 thymocytes compared to DN3 cells but the difference in Rag2 expression in the adult thymocyte population was not significant (Fig. 3A, right panel). When foetal and adult Rag1^–/–^ thymocytes were treated with antiCD3 antibody for 24 h to mimic the pre-TCR signal, down-regulation of Rag2 transcription was even greater (6–12-fold) compared to down-regulation of Rag2 in foetal DN4 wild-type thymocytes (Fig. 3B). The latter observation suggests that, although pre-TCR was not necessary for down-regulation of the Rag genes at the DN4 stage, a strong signal can accelerate down-regulation of Rag2.

### D to J TCRβ rearrangement in TCRβ^–^/γδ^–^ DN4 thymocytes

To further assess the origin and lineage commitment of the TCRβ^–^/γδ^–^ DN4 cells we analysed the extent of their DJ TCRβ rearrangement. We sorted DN4 TCRβ^–^/γδ^–^ and TCRβ/γδ^+^ cells from E16.5 foetal wild-type thymi (Fig. 3C, D) and performed a PCR-Southern assay to compare the extend of DJ TCRβ rearrangement in the two cell types, as previously described [17]. We amplified the TCRβ locus from equal amounts of DNA template from sorted TCRβ^–^/γδ^–^ and TCRβ/γδ^+^ DN4 cells using Dβ2-and Jβ2.7-specific primers. We observed similar level of DJ rearrangement in both cell types (Fig. 3F, G) demonstrating that the TCRβ^–^/γδ^–^ DN4 cells are T lineage committed and suggesting that they originate from DN2/DN3 cells.

### TCRβ expression promotes proliferation in foetal DN4 thymocytes

**Figure 4:**
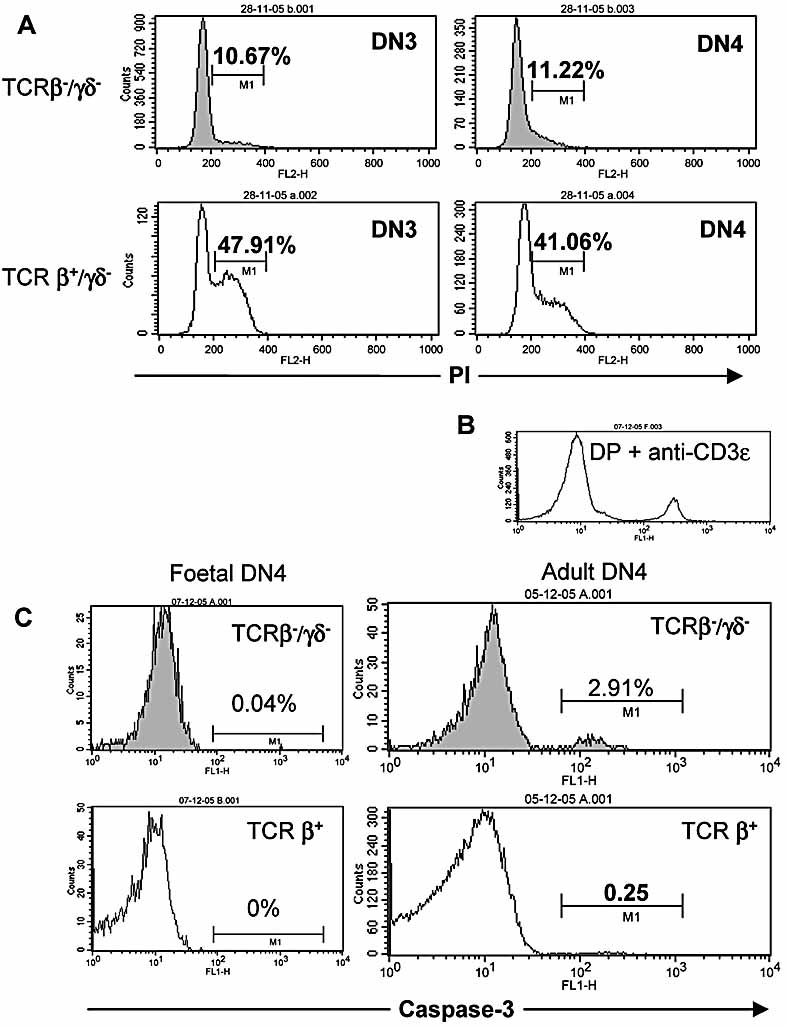
Proliferation and apoptosis of wild-type foetal thymocytes. (A) DNA content analysis by PI nuclear staining of sorted CD44^–^CD3^–^CD4^–^CD8^–^B220^–^Thy1.2^+^ TCRβ^–^/γδ^–^ CD25^+^ (DN3, top left histogram) and CD25^–^ (DN4, top right histogram) and TCRβ^+^ DN3 (bottom left histogram) and DN4 (bottom right histogram) cells. Percentages of cells in the S/G2/M phases of the cell cycle are shown. (B) Intracellular expression of the active form of caspase-3 in DP cells where apoptosis was induced by incubation with anti-CD3 antibody for 2 h. (C) Intracellular expression of the active form of caspase-3 in foetal and adult DN4 thymocytes. Histograms on the left show active caspase-3 expression in TCRβ^–^/γδ^–^ (top) and TCRβ^+^ (bottom) E16.5 foetal CD44^–^CD25^–^CD3^–^CD4^–^CD8^–^B220^–^ Thy1.2^+^ (DN4) thymocytes and histograms on the right show active caspase-3 expression in TCRβ^–^/γδ^–^ (top) and TCRβ^+^ adult DN4 populations. Percentages of active caspase-3 positive cells are shown.

A gradual decrease in the proportion of icTCRβ^–^ DN4 cells during embryogenesis suggests that the foetal DN4 TCRβ^+^ subset expands faster than the DN4 TCRβ^–^ subset. To test whether this is due to increased proliferation, we sorted E16.5 foetal DN3 and DN4 cells based on the intracellular expression of β and γδ TCR and analysed proliferation. In a typical experiment, propidium iodide DNA staining showed that approximately four-times more TCRβ^+^ DN3 (47.91%) and DN4 (41.06%) cells were in cell cycle than in the TCRβ^–^/γδ^–^ DN3 (10.67%) or DN4 (11.22%) subset (Fig. 4A), consistent with a pre-TCR induced signal for proliferation [5, 14, 18–21].

We also tested the possibility that the percentage of DN4 TCRβ^–^/γδ^–^ cells decreased due to active apoptosis in this subset. We analysed the intracellular expression of the active form of caspase-3, as it has recently been shown that pre-TCR up-regulates the anti-apoptotic gene BCL2A1 that rescues cells from caspase-3-mediated apoptosis [9]. We were able to detect induction of apoptosis in control, adult DP cells treated for 2 h with antiCD3 (Fig. 4B), but the active form of caspase-3 was virtually absent in both icTCRβ^–^/γδ^–^ and icTCRβ^+^ foetal DN4 thymocytes (Fig. 4C) and among DN3 thymocytes (data not shown). In contrast in the adult thymus we were able to detect more apoptotic cells amongst the icTCRβ^–^/γδ^–^ DN4 subset compared to TCRβ^+^ DN4 subset. In a typical experiment 2.91% of icTCRβ^–^/γδ^–^ DN4 cells were caspase-3 positive compared to 0.25% of the icTCRβ^+^ DN4 cells (Fig. 4C). Our data indicate that in the foetal thymus, clonotypic TCRβ expression promotes proliferation of DN thymocytes. Lack, however, of TCRβ expression did not immediately induce cell death in foetal DN4 cells, as evidenced by the absence of active caspase-3-expressing cells in the DN4 icTCRβ^–^/γδ^–^ population. The increased apoptosis that we observed in the adult DN4 icTCRβ^–^/γδ^–^ population might be attributed to the accumulation of ageing cells in this subset.

### Abundance of adult TCRβ^−^/γδ^−^ DN4 cells after hydrocortisone treatment

**Figure 5:**
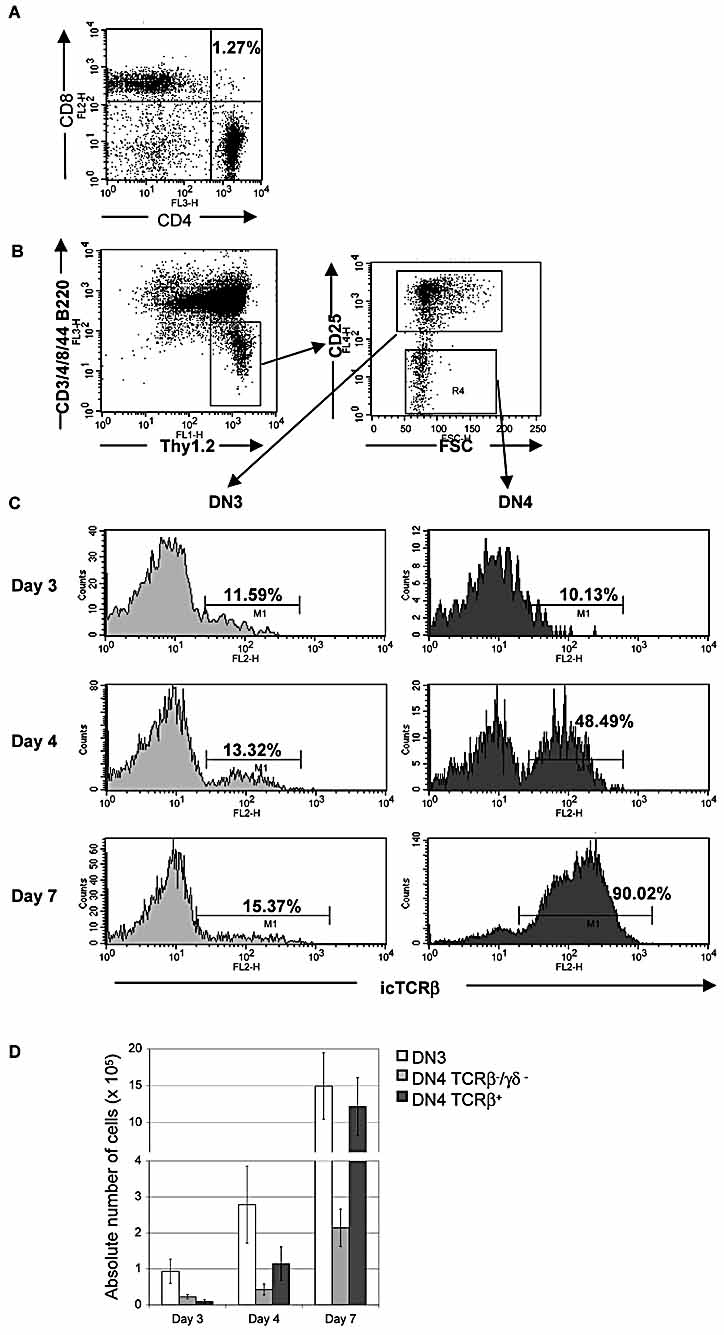
Expression of TCRβ in thymocytes that differentiated following hydrocortisone treatment. (A) CD4 and CD8 expression profiles of HC-treated thymus 3 days after treatment. (B) Identification of DN3 and DN4 subsets. Cells were selected to be negative for CD3, CD4, CD8, CD44 and B220 and positive for Thy1.2 expression (left). DN3 cells were selected positive for CD25 expression and DN4 cells negative for CD25 expression (right). Representative dot plots are shown for thymocytes prepared 3 days after HC treatment. (C) Intracellular expression of TCRβ in DN3 (lighter shaded histograms) and DN4 (darker shaded histograms) populations 3, 4 and 7 days after HC treatment. Percentages of icTCRβ^+^ cells are shown. (D) Graph representing the kinetics of DN3, DN4 TCRβ^–^/γδ^–^ and DN4 TCRβ^+^ cell numbers in the HC-treated mice. Open bars represent the absolute number of DN3 thymocytes, light shaded bars of DN4 TCRβ^–^/γδ^–^ thymocytes and darker bars of DN4 TCRβ^+^ thymocytes 3, 4 and 7 days after HC treatment. Mean thymocyte numbers were at day 3 (0.43 ± 0.06) × 10^7^, day 4 (0.78 ± 0.14) × 10^7^ and day 7 (6.92 ± 1.65) × 10^7^. Mean DN4 TCRβ^–^/γδ^–^ thymocyte numbers were at day 3 (0.23 ± 0.06) × 10^5^, at day 4 (0.43 ± 0.15) × 10^5^ and at day 7 (2.15 ± 0.52) × 10^5^.

Differentiation of foetal thymocytes occurs in a largely synchronized wave. In adults, however, thymocyte production is continuous and the adult thymus has reached steady state with pre-existing cells at all stages of development and homeostasis between thymocyte populations. The difference in the expression of TCRβ in the DN4 subset that we observe between foetal and adult thymocytes could be due to a foetal restricted capability of thymocytes to differentiate to the DN4 stage independently of normal pre-TCR signalling, either as a result of differences between foetal and adult hematopoietic progenitors [22, 23] or between the foetal and adult thymic microenvironment [24]. Alternatively, it is possible that adult TCRβ/γδ^–^ thymocytes differentiate to the DN4 stage as efficiently as foetal thymocytes but that their percentage is very low due to the pre-existing high number of proliferating TCRβ^+^ DN4 thymocytes that mask their presence. To distinguish between these two hypotheses, we synchronized the differentiation of immature thymocytes by treating adult mice with hydrocortisone (HC). HC treatment induces apoptosis of immature DP thymocytes [25] but mature single positive cells survive treatment and the thymus then grows exponentially to recover its normal size and subset distribution a week following the treatment. HC enabled us to study the differentiation of thymocytes in the adult thymus in a synchronized wave and in a rapidly expanding thymus that had not yet reached homeostasis. At 3 days after HC treatment, the thymus contained only 1.27% DP thymocytes (Fig. 5A). Analysis of intracellular expression of TCRβ in DN3 and DN4 cells, identified by the absence of surface expression of CD3, CD4, CD8, CD44 and B220 markers, the presence of Thy1.2, and presence or absence, respectively, of CD25 (Fig. 5B) showed that the percentage of cells expressing TCRβ was equivalent between DN3 (11.59%) and DN4 (10.13%) populations (Fig. 5C, top panel). At 4 days after HC treatment, the percentage of icTCRβ^+^ cells in the DN4 population significantly increased to 48.49% compared to 13.32% of DN3 cells (Fig. 5C, middle panel). At 7 days after treatment, the percentage of TCRβ^+^ DN4 cells had reverted back to the percentage observed in non-treated wild-type adult mice with typically 15.37% of DN3 and 90.02% of DN4 cells staining positive for icTCRβ^+^ (Fig. 5C, lower panel), and 90.36% of thymocytes were CD4^+^CD8^+^DP, indicating that the thymus had fully recovered (data not shown). Although the absolute number of DN3, DN4 TCRβ^–^/γδ^–^ and DN4 TCRβ^+^ cells increased overtime, there was preferential expansion of the DN3 and TCRβ^+^ DN4 populations (Fig. 5D).

**Figure 6:**
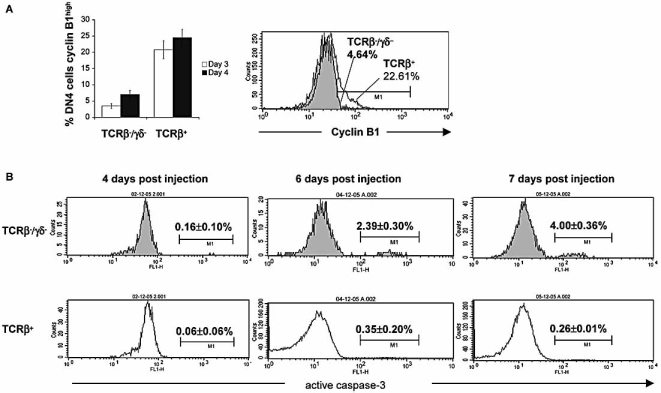
Proliferation and apoptosis in DN4 population after HC treatment. (A) The left panel is the percentages of icTCRβ^–^/γδ^–^ and icTCRβ^+^ DN4 cells positive for high level of intracellular expression of cyclin B1, indicative of cells at the S/G2/M cell cycle stages. Bars represent the mean of five mice analysed 3 days (open bars) and 4 days (black bars) after HC treatment. The right panel is representative histogram overlay of intracellular cyclin B1 staining of icTCRβ^–^/γδ^–^ (full histogram) and icTCRβ^+^ (empty histogram) DN4 cells 4 days after HC treatment. (B) Intracellular expression of the active form of caspase-3 in DN4 thymocytes after HC treatment. Histograms show caspase-3 expression in TCRβ^–^/γδ^–^ (shaded histograms) and TCRβ^+^ (open histograms) DN4 thymocytes after 4 days (left), 6 days (middle) and 7 days (right). Percentages of active caspase-3-positive cells are shown.

To assess cell cycle status in the DN4 population, we compared cyclin B1 expression in the TCRβ^–^/γδ^–^ and TCRβ^+^ DN4 thymocytes at 3 and 4 days after HC treatment (Fig. 6A). Approximately four times more cells in the TCRβ^+^ DN4 compartment showed high intensity cyclin B1 staining (Fig. 6A), indicative of cells in the S/G2/M stages of the cell cycle, than in the TCRβ^–^/γδ^–^ DN4 population, indicating that TCRβ expression (and hence pre-TCR signalling) induces expansion.

We also analysed apoptosis of the DN4 thymocytes 4, 6 and 7 days after HC treatment by caspase-3 staining. Apoptosis was virtually undetectable 4 days after treatment in both TCRβ^–^/γδ^–^ (0.16%) and TCRβ^+^ (0.06%) DN4 cells (Fig. 6B, left). In contrast, at 6 days after treatment, the percentage of apoptotic cells was significantly higher in TCRβ^–^/γδ^–^ DN4 cells (2.39%) than in TCRβ^+^ DN4 cells (0.35%, Fig. 6B, middle) and had further increased 7 days after treatment to 4%, whereas no increase in apoptosis was observed in the TCRβ^+^ DN4 subset (0.26%, Fig. 6B, right).

In summary, in the synchronised and rapidly expanding HC-treated adult thymus, we were able to detect a large percentage of TCRβ^–^/γδ^–^ adult DN4 thymocytes, similar to that observed in the foetal thymus. Thus, the rarity of TCRβ^–^/γδ^–^ DN4 cells in the normal adult thymus cannot be attributed to an intrinsic inability of either adult lymphoid or stromal compartments to support pre-TCR independent differentiation to DN4. Our experiments suggest that the decrease in the ratio of TCRβ^–^/γδ^–^:TCRβ^+^ DN4 cells after treatment is due to pre-TCR induced expansion of the TCRβ^+^ subset rather than apoptosis of the TCRβ^–^/γδ^–^ DN4 population. The detection of significantly more apoptotic cells in the TCRβ^–^/γδ^–^ DN4 subset in non-treated adults and 7 days after HC treatment seems to reflect ageing cells accumulating in this subset rather than immediate induction of apoptosis due to lack of pre-TCR signalling.

### Reduced proliferation, increased apoptosis in foetal Rag1^–/–^ DN4 *versus* DN3

**Figure 7:**
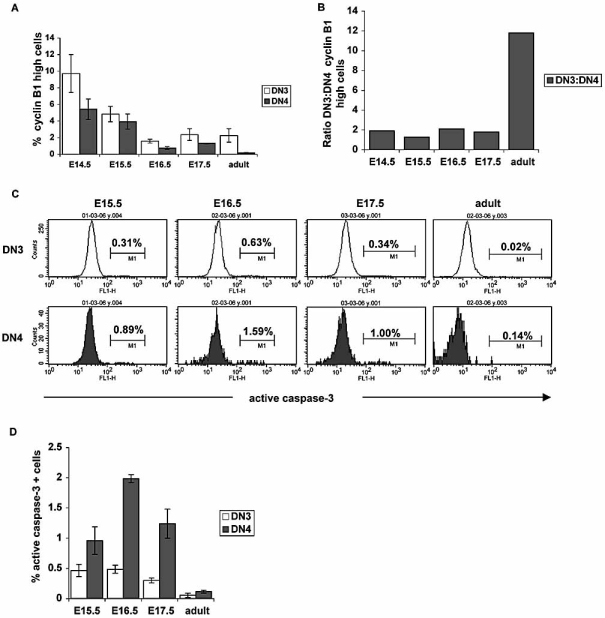
Proliferation and apoptosis of Rag1^–/–^ foetal and adult DN thymocytes. (A) Percentages of DN3 (open bars) and DN4 (shaded bars) Rag1^–/–^ cells positive for high expression of cyclin B1 in thymi at E14.5, E15.5, E16.5 and, E17.5 and adult thymus. Bars represent data from at least three animals and error bars are shown. (B) Ratio of proliferation of DN3 relative to DN4 cells as in (A). (C) Intracellular expression of the active form of caspase-3 in DN3 (open histograms) and DN4 (shaded histograms) subsets of Rag1^–/–^ E15.5, E16.5, E17.5 and adult thymocytes. Percentages of caspase-3-positive cells are shown. (D) Mean percentages of active caspase-3-positive DN3 (open bars) and DN4 (shaded bars) E15.5, E16.5, E17.5 and adult thymocytes. Bars represent data from at least three animals and error bars are shown.

To account for the virtual absence of DN4 cells in Rag1^–/–^ adult thymus compared to the readily detectable population in the Rag1^–/–^ foetal thymus, we analysed proliferation and apoptosis of Rag1^–/–^ DN3 and DN4 cells during embryonic development and in the adult Rag1^–/–^ thymus. On all embryonic days tested the percentage of DN4 cells that were in cell cycle, as assessed by the intensity of cyclin B1 staining, was lower than the corresponding percentage of DN3 cells, and the percentage of cycling cells decreased over time in both DN3 and DN4 subsets (Fig. 7A). Interestingly, the ratio of cycling cells in the DN3 relative to the DN4 population increased sixfold between foetal and adult thymus, accounting for the decline in the DN4 population (Fig. 7B).

Staining for the active form of caspase-3 revealed that, contrary to the data obtained from the wild-type foetal thymus, apoptotic cells were present in Rag1^–/–^ foetal DN3 and DN4 subsets and their percentage was higher in the DN4 subset (Fig. 7C, D). In a typical experiment the percentage of apoptotic cells in the DN3 subset varied from 0.31% and 0.63% for E15.5–17.5 and from 0.89% to 1.58% in the DN4 subset (Fig. 7C). In the adult Rag1^–/–^ thymus, we were only able to detect a very small percentage of active caspase-3-positive cells in both the DN3 (0.02%) and the DN4 (0.14%) subsets (Fig. 7C) and there was no significant difference in the percentage of apoptotic cells between the two subsets (Fig. 7D). The observed increase in apoptosis in the foetal DN4 subset relative to the DN3 subset during ontogeny provides an additional explanation for the decline of the DN4 population.

## Discussion

Here we showed that in the foetal thymus down-regulation of CD25 cell surface expression and differentiation to the DN4 stage can occur independently of TCRβ expression and hence pre-TCR signalling. Rare TCRβ^–^/γδ^–^ DN4 cells in adult mice have been previously reported [7, 15], but we showed that in contrast to the adult thymus, most DN4 thymocytes in the E15.5 foetal thymus were icTCRβ^–^/γδ^–^. These TCRβ^–^/γδ^–^ CD25^–^CD44^–^ thymocytes were T lineage, based on expression of Thy1 and icCD3, and TCRβ DJ rearrangement. Likewise, we found a normal percentage of DN4 cells in the early Rag1^–/–^ foetal thymus, despite their inherent inability for TCRβ/γδ rearrangement and expression.

Most DN4 cells that emerged after HC treatment of adult mice were also icTCRβ^–^/γδ^–^. The abundance of TCRβ^–^/γδ^–^ DN4 cells in the HC-treated adult thymus demonstrated that their production was not foetal-restricted and that both adult lymphoid and stromal elements are capable of supporting differentiation to DN4 in the absence of TCRβ/γδ expression. This similarity with the foetal thymus could reflect the synchronicity of the transition in both cases (enabling the identification of normally undetectable TCRβ^–^/γδ^–^ that would physiologically make the transition from DN3 to DN4 in the adult thymus). Alternatively, the production of TCRβ^–^/γδ^–^ DN4 cells in the adult HC-treated thymus could be a direct consequence of the treatment, as regeneration and rapid growth are induced. During embryogenesis, the foetal thymus also expands exponentially. It therefore remains to be determined to what extent pre-TCR independent mechanisms of differentiation from DN3 to DN4 occur in the adult thymus under physiological conditions, but clearly TCRβ/γδ expression independent differentiation from DN3 to DN4 is important in the foetal thymus. It likewise remains to be determined if low-level signalling through other components of the pre-TCR complex in the absence of TCRβ expression, such as CD3 polypeptides, would be sufficient to drive down-regulation of CD25.

The fact that DN4 thymocytes are found in the foetal Rag1^–/–^ thymus is important for the interpretation of the phenotype of other genetically altered mice that have arrested thymocyte development. For example, particularly in the foetus, the appearance of DN4 cells may have lead to the assumption that a mutation affected processes following pre-TCR signalling when in fact this might not necessarily be the case.

There are several studies indicating that pre-TCR signalling and β-selection induce proliferation [5, 14, 18–21]. We also demonstrated directly, both by PI and anti-cyclin B1 staining that four times more TCRβ^+^ cells were dividing in both the foetal and HC-treated adult thymus, compared to the TCRβ^–^/γδ^–^ DN4 population. We found more proliferating cells in both foetal TCRβ^+^ and TCRβ^–^/γδ^–^ DN4 cells than in their adult counterparts, consistent with the exponential increase in thymocyte number during embryogenesis. In addition, it is worth noting that we detected some cell division in all thymocyte populations examined, including the TCRβ^–^/γδ^–^ DN4 population, suggesting the existence of pre-TCR independent mechanisms of proliferation, as previously reported [7, 26].

It has been shown that the pre-TCR can promote cell survival by induction of anti-apoptotic agents [9] and that cell death is increase in the adult TCRβ^–^ DN4 population relative to the adult TCRβ^+^ DN4 [7]. The experiments of Falk and others [7] correlated cell surface CD69 expression with absence of icTCRβ/γδ in the DN4 population, and then demonstrated relatively more apoptosis in the CD69^+^ DN4 population than in the CD69^–^ DN4 cells. Consistent with this study, our analysis confirms directly an increase in apoptosis of TCRβ^–^/γδ^–^ DN4 cells relative to TCRβ^+^ DN4 cells in the adult thymus (Fig. 4C). Our experiments suggest, however, that absence of the pre-TCR complex does not immediately induce apoptosis as we did not observe apoptosis amongst the foetal or recently differentiated adult DN4 cells either by caspase-3 or annexin-V staining (Fig. 4 and 6, and data not shown), regardless of the presence or absence of TCRβ/γδ. The increase in apoptosis in the TCRβ^–^/γδ^–^ DN4 population on successive days after HC treatment is consistent with accumulation of ageing cells and absence of pre-TCR induced pro-survival signals that are up-regulated in the TCRβ^+^ DN4 population [9]. The proportion but not the number of icTCRβ^–^γδ^–^ DN4 cells greatly decreased over time, and was very low in the fully developed adult thymus. Taken together our experiments indicate that the age-dependent decline in the proportion of TCRβ^–^ DN4 cells is due to pre-TCR induced proliferation of the TCRβ^+^ DN4 subset, rather than immediate induction of apoptosis in cells that fail to pass the β-selection check-point at the DN3 stage.

The fate of the TCRβ^–^/γδ^–^ DN4 cells is unclear. In the foetal and HC-treated adult thymus, there was no enrichment for TCRβ^+^ cells in the DN4 subset compared to the DN3 population. It is possible that some wild-type TCRβ^–^/γδ^–^ DN4 cells eventually rearrange and express TCRβ or TCRγδ and continue their maturation, but the foetal Rag1^–/–^ DN4 thymocytes clearly cannot progress beyond the DN4 stage as neither DP nor SP cells are observed in either the foetal or adult Rag1^–/–^ thymi. We showed that the foetal Rag1^–/–^ DN4 population is disadvantaged compared to the DN3 population due to both reduced proliferation and increased apoptosis, explaining the virtual absence of DN4 cells in the adult thymus. In addition, the presence of apoptotic Rag1^–/–^ DN3 and DN4 cells, not detectable in the equivalent wild-type populations and the reduced proliferation in the Rag1^–/–^ foetal compared to wild-type thymocyte populations seem to reflect differences in the thymic microenvironment of Rag1^–/–^ and wild-type thymus. This is consistent with a previous report showing that the reduced proliferation of adult Rag2^–/–^ compared to wild-type thymocytes was at least partly due to differences in the thymus environment rather than the absence of cell autonomous, pre-TCR related proliferation signals in the Rag2^–/–^ thymocytes [26].

In conclusion, we have shown that in the foetal thymus the transition from the DN3 to the DN4 stage is frequently independent of pre-TCR signalling. Our data also suggest that pre-TCR signalling induces proliferation and that lack of it does not immediately induce apoptosis.

## Materials and methods

### Mice

Wild-type mice were C57BL/6, purchased from B & K Universal Ltd (UK) and Rag1^–/–^ [10] mice were purchased from Jackson Laboratories, USA. All mice were bred and maintained at the Central Biomedical Services unit at Imperial College London according to UK Home Office regulations.

Timed mates were performed by mating a male with two females overnight and monitoring the females for plugs. The day the plug was found was counted as E0.5.

### Flow cytometry and antibodies

Thymocyte suspensions were prepared by crushing thymi between two pieces of ground glass. Cells were stained using combinations of directly conjugated antibodies obtained from BD Pharmingen: anti-CD44^FITC^, anti-CD44^PE^, anti-CD44^Cychrome^, anti-CD25 ^PE^, anti-CD25 ^FITC^, anti-CD4^FITC^, anti-CD4^PE^, anti-CD4^Cychrome^, anti-CD8 α ^FITC^, anti-CD8 α ^PE^, anti-CD8α ^Cychrome^, anti-CD3 ^Cychrome^, anti-CD45.2^FITC^, anti-Thy1.2^FITC^, anti-HSA^PE^, anti-NK1.1^FITC^, anti-B220^Cychrome^, anti-TCRβ^PE^, and anti-TCRγδ^PE^.

Cell suspensions were stained with the antibodies for 30 min on ice in 50 μl Dulbecco's modified medium (Life Technologies), supplemented with 5% FCS and 0.01% sodium azide. Cells were washed in this medium between incubations and prior to analysis on the FACScan (Becton Dickinson). Events were collected in list mode using CellQuest software and data analysed using CellQuest Pro software. Live cells were gated according to their FSC and SSC profiles. Data are representative of at least three experiments.

Intracellular staining for TCRβ, TCRγ/δ, CD3, cyclin B1 and caspase-3 was performed on cells stained for surface markers as above following fixation and permeabilisation with the Cytofix/Cytoperm™ solutions (BD biosciences) according to the manufacturer's instructions.

PI (Sigma) staining was carried out on sorted thymocytes treated with 100 μg/mL RNase (Sigma) and permeabilised in 0.1% Triton X-100, as described previously [27]. Cell cycle analysis was carried out using a doublet-discrimination module on the FACScan.

C57BL/6J E15.5 and E16.5 foetal and adult thymocytes were sorted on a Modular Flow Cytometer (MoFlo, Cytomation, Inc., Fort Collins, CO) at the Cancer Research UK FACS laboratory. For purification of DN3 and DN4 populations for the real time RT-PCR analysis of Rag1 and Rag2 transcription, cells falling within the FSC/SSC live gate, >98% of which were CD45.2^+^, were sorted using antibodies directed against CD25^FITC^, CD3/CD4/CD8^PE^ and CD44^Cychrome^. TCRβ^–^/γδ^–^ and TCRβ^+^ DN4 thymocytes were isolated as described in Fig. 3C, D, falling within the FSC/SSC live gate and staining negative for CD3/CD4/CD8/CD25/CD44/B220 and positive for Thy1.2 and negative or positive for icTCRβ/γδ or icTCRβ antibody staining, respectively.

### Foetal and adult thymus organ cultures

Foetal and adult thymi were cultured on 8-μm pore size Millipore filters (Millipore) in AIM-V serum free medium (Life Technologies) at 37°C and 5% CO_2_ with 1 μg/mLazide-freeanti-CD3 (BD Pharmingen) as previously described [17].

### Real-time RT-PCR analysis

RNA was extracted from the sorted thymocytes with the Absolutely RNA miniprep kit (Stratagene) and cDNA was synthesized with Superscript III (Invitrogen, Carlsbad, CA). The cDNA samples were analysed in triplicate by real-time PCR on an iCycler (Bio-Rad Laboratories) using the iQ^TM^SYBR® Green Supermix (Bio-Rad) according to the manufacturer's instructions and as previously described [17]. At least one primer for each amplification pair was designed to span exon-exon boundaries to avoid amplification of genomic DNA. For the amplification of *Rag1* transcripts, we used primers Rag1 exon 2 CCTTTGAGGTTTCTCTAAC and Rag1 exon 2–3 TCCTGCTACTAAAATCTCC, and for the amplification of *Rag2* transcripts we used Rag2 forward CTGGCTTGGCCGAAAGG and Rag2 reverse CTGCTTGTGGATGTGAAATACTCT.

### Quantification of TCRβ locus rearrangements

TCRβ^–^/γδ^–^ and TCRβ/γδ^+^ DN4 thymocytes were sorted from E16.5 foetal thymi as described above, DNA was extracted using the DNeasy tissue kit (Qiagen) and D to J PCR-Southern hybridisation assay was carried out as previously described [17]. In brief, 24 cycles of PCR amplification for the D to J TCRβ rearrangements were performed using the following primers: Dβ2 (5′) GTAGGCACCTGTGGGGAAGAAACT and Jβ2.7 (3′) TGAGAGCTGTCTCCTACTATCGATT, and the amplified products were quantified by Southern blotting using a TCRβ probe amplified from germline (unrearranged) TCRβ locus with Dβ2 and Jβ2.7 primers, corresponding to the genomic region between the TCRDβ2 and TCRJβ2.7 as previously described [17].

The genomic DNA concentration of the templates for the D to J PCR-Southern assay were quantified by real time PCR on an iCycler using primers specific for a single copy genomic locus 5′G2FAGAACCTGAAGACACACCTGCG and 3′G2R GAGGCATTGGAGAAGGCTTTG.

### HC treatment

For the deletion of immature thymocytes and synchronization of thymocyte development in adult thymus, 4–8-week-old C57BL/6 mice were injected intraperitoneally with 0.4 mg/g HC sodium phosphate (Sigma), diluted in sterile PBS [25].

## Abbreviations:

DN: Double negative
DP: double positive
E: embryonic day
FTOC: foetal thymus organ culture
HC: hydrocortisone
ic: intracellular
